# Interaction between Mesenchymal Stem Cells and B-Cells

**DOI:** 10.3390/ijms17050650

**Published:** 2016-05-05

**Authors:** Linxiao Fan, Chenxia Hu, Jiajia Chen, Panpan Cen, Jie Wang, Lanjuan Li

**Affiliations:** Collaborative Innovation Center for Diagnosis and Treatment of Infectious Diseases, State Key Laboratory for Diagnosis and Treatment of Infectious Diseases, School of Medicine, First Affiliated Hospital, Zhejiang University, Hangzhou 310003, China; 21418049@zju.edu.cn (L.F.); 11318093@zju.edu.cn (C.H.); jiajiatale@163.com (J.C.); cenpanpan90@163.com (P.C.); wj498624370@126.com (J.W.)

**Keywords:** mesenchymal stem cell, B-cell, immune regulation, immune suppression, systemic lupus erythematous, graft-versus-host disease, T-cell, plasma cell, cytokine

## Abstract

Mesenchymal stem cells (MSCs) are multipotent; non-hematopoietic stem cells. Because of their immunoregulatory abilities; MSCs are widely used for different clinical applications. Compared with that of other immune cells; the investigation of how MSCs specifically regulate B-cells has been superficial and insufficient. In addition; the few experimental studies on this regulation are often contradictory. In this review; we summarize the various interactions between different types or states of MSCs and B-cells; address how different types of MSCs and B-cells affect this interaction and examine how other immune cells influence the regulation of B-cells by MSCs. Finally; we hypothesize why there are conflicting results on the interaction between MSCs and B-cells in the literature.

## 1. Introduction

Mesenchymal stem cells (MSCs) are a progenitor cell population with multilineage potency. MSCs were initially discovered in bone marrow and were subsequently found in almost every type of tissue [1,2,3], including adipose tissue [4], the placenta [5], umbilical cord [6], endometrium [7], and gingiva [8]. When cultured *in vitro*, MSCs are able to proliferate, form plastic-adherent colonies and retain the capability to perform osteogenesis, chondrogenesis, and adipogenesis [9]. These cells also have multilineage potency *in vivo* and are able to generate functional cells for use in regenerative medicine. However, the research focus has recently shifted, with a new appreciation of the wide range of MSC-secreted trophic factors that are capable of promoting tissue repair and potent immune modulation [1]. Recent evidence suggests that MSCs can regulate T-cells [6,10], natural killer cells (NK-cells) [11], dendritic cells (DCs) [12], and macrophages [13]. A remarkable curative effect can be observed in the treatment of systemic lupus erythematous (SLE) [6], graft-versus-host disease (GVHD) [14], type I diabetes [4], inflammatory bowel disease (IBD) [8], and pancreatic islets transplantation [15]. Compared with the clear mechanism of interaction between MSCs and the immune cells mentioned above, the investigation of the immune regulation of B-cells by MSCs has been superficial and insufficient, and the results are commonly contradictory between different experimental studies [16,17].

B-cells, a type of lymphocyte, are indispensable for the humoral immunity portion of the human adaptive immune system. B-cells secrete antibodies (when stimulated by antigens), present antigens and secrete cytokines, such as interleukin-10 (IL-10) [18,19]. B-cells develop from hematopoietic progenitor cells in the fetal liver and, after birth, in the bone marrow [20,21]. The development, proliferation, differentiation and maturation of B-cells are all complex and sophisticated controlled processes *in vivo*. Cytokines (IL-7 [22], IL-6 [23], IL-4 [24], and interferon-γ (IFN-γ) [24], *etc.*) and other types of lymphocytes (T-cells [25], DCs [26] and macrophages [26]) play a guiding role in regulating the B-cell behaviors mentioned above. There is also a considerable amount of MSCs in the bone marrow [3]. The presence of MSCs in both the fetal and adult liver has also been confirmed [27,28]. The presence of MSCs in the sites of B-cell development and maturation is significant. Autoimmune diseases, including SLE, multiple sclerosis and type 1 diabetes, can arise when abnormal B-cells recognize self-antigens and secrete autoantibodies [29].

Previous reports have found that MSCs can alter the functions of immune cells with both cell-to-cell contact and the secretion of soluble cytokines, including IL-10 [30], transforming growth factor-β (TGF-β) [31], prostaglandin E2 (PGE2) [10], nitric oxide (NO) [32], and indoleamine 2,3,dioxygenase (IDO) [33]. These cytokines can influence the interaction between MSCs and other immune cells. However, for B-cells, studies currently suggest that the response of B-cells to MSCs depends on the culture microenvironment. Thus, although B-cells may directly interact with MSCs to alter their characteristics and behaviors, other immune cells (e.g., T-cells and DCs) also play an indirect regulatory role and may serve as intermediates between the MSCs and B-cells. After reviewing the literature, we cannot yet arrive at general conclusion regarding the interaction between various MSC and B-cell types and their activation states. However, it is clear that the interaction between MSCs and B-cells is extremely complicated and is likely associated with many different factors. After the mechanisms of interaction between MSCs and B-cells and subsequent immune regulation are clarified, manipulation of this interaction may lead to effective therapies in tissue engineering and regenerative medicine. In this review, we focus on the interaction between MSCs and B-cells in purified B-cell co-culture systems, as well as in multi-immune cell co-culture systems, to clarify the underlining mechanisms of their interaction.

## 2. Characterization of MSC-B Cells Interactions

Although MSC/purified B-cell (MSC–B) co-culture conditions are not identical to the conditions that occur *in vivo*, MSC–B is the optimal controllable system for investigating the interaction between MSCs and B-cells. There are few interfering factors in this system; thus, the experimental results are repeatable and interpretable. Experiments using the MSC–B system will lay a solid foundation for further studies. The culture medium, cell origin of the MSCs and B-cells, and the maturity of the B-cells used in any given experiment will likely influence the interaction between MSCs and B-cells.

### 2.1. Different Origins and Activation States of MSCs

MSCs isolated from different origins, or those in different states of activation, can regulate the survival or proliferation of B-cells differently. For example, MSCs from lupus patients (LMSCs) are unable to regulate the immune system, causing symptoms and pathological changes in SLE [34]. To examine the mechanism underlying this defect, a LMSC–B-cell co-culture system was designed. The LMSCs were able to promote levels of proliferation of unstimulated B-cells similar to those of MSCs from healthy individuals [16]. However, LMSCs were less able to inhibit the proliferation, plasma cell differentiation, and immunoglobulin M and immunoglobulin G (IgM and IgG) secretion of B-cells that were stimulated by a stimulation cocktail (cytosine phosphorothioate (CpG), soluble cluster of differentiation 40 ligand (sCD40L), antiIgM, and IL-4) compared with MSCs from healthy subjects [16,35]. The expression level of C–C-motif ligand 2 (CCL2), which can be cleaved by matrix metalloproteinases (MMPs), is also lower in LMSCs, likely because MMP-1 is able to mediate the suppression of B-cells by MSCs [36]. In contrast, overexpression of olfactory 1/early B-cell factor-associated zinc-finger protein (OAZ) in LMSCs can downregulate CCL2 [36].

CCAAT/enhancer-binding protein β (C/EBPβ), a protein expressed on the cytomembrane of bone marrow mesenchymal stem cells (BMMSCs), can promote the proliferation and differentiation of B-cells. C/EBPβ-deficient BMMSCs have an impaired ability to support the differentiation of hematopoietic stem cells (HSCs) into precursor B-cells, and the reduced production of C–X–C-motif ligand (CXCL12)/stromal cell-derived factor 1 (SDF-1) by C/EBPβ-deficient BMMSCs partially contributes to this phenotype. Furthermore, the survival of leukemic precursor B-cells is also suppressed when these cells are co-cultured with C/EBPβ-deficient BMMSCs [37]. Supernatant extracts from MSCs differentiated into adipocytes (adi-MSCs) can promote the proliferation of activated B-cells in a dose-dependent manner. This effect is mainly attributed to B-cell activating factor (BAFF) secretion by adi-MSCs, whereas undifferentiated MSCs show the opposite effect on B-cells [38]. In MSCs, Toll-like receptor 4 (TLR4) can upregulate BAFF, which in turn promotes the nuclear factor kappa-light-chain-enhancer of activated B-cells (NF-κB), c-Jun N-terminal kinases (JNK), and p38 mitogen-activated protein kinase (MAPK) signaling pathways. In addition, activated TLR4 can promote either MSC osteogenic or adipogenic differentiation, suggesting that the stimulation of B-cell proliferation by TLR4 and MSCs is highly correlated [39]. According to Nan Che *et al.*, umbilical cord MSCs (UCMSCs) can inhibit the proliferation and plasma cell differentiation of B-cells, whereas IgM and IgG secretion is suppressed. In addition, when B-cells were co-cultured with UCMSCs, the expression of B-cell-induced maturation protein-1 (Blimp-1) was downregulated, whereas the expression of paired box gene 5 (PAX-5) was upregulated, and the phosphorylation of the phosphorylated protein-38 (p-p38) and phosphorylated protein kinase B (p-PKB) pathways were inhibited [40]. According to Charalampos Pontikoglou *et al.*, BMMSCs from B-cell chronic lymphoblastic leukemia patients (CLLBMMSCs) can promote the proliferation and IgG secretion of B-cells, whereas healthy BMMSCs do not promote proliferation or IgG secretion. CLLBMMSCs are also able to regulate B-cell apoptosis, unlike healthy BMMSCs. CLLBMMSCs have been shown to secrete more proliferation-inducing ligand (APRIL) and less SDF-1, BAFF, and TGF-β1 than healthy BMMSCs [41]. Additionally, MSCs infected with *Mycoplasma arginini* show increased inhibitory effects on the Ig production of IL-4/lipopolysaccharide (LPS)-stimulated B-cells compared with mycoplasma-free MSCs. Complement C3 (C3) has also been shown to be involved in the suppression of B-cell Ig production by infected MSCs. In this process, Blimp-1 may be inactivated directly or indirectly by infected MSCs [42].

Despite varying the origin or culture medium, MSCs activated by IFN-γ or tumor necrosis factor-α (TNF-α) inhibit B-cell proliferation, whereas unstimulated MSCs do not suppress B-cell proliferation and may even promote proliferation to some extent. In either amesenchymal stem cell from adipose tissue (ASC)–human platelet lysate (PL) system or a BMMSC–fetal calf serum (FCS) system [16], BMMSCs stimulated by TNF-α inhibited the release of IgE and IgG from activated B-cells but had no effect on B-cell survival. The cyclo-oxygen-ase 2(COX2)/PGE2 signaling pathway may play a key role mediating this inhibition [43]. MSCs stimulated by IFN-γ can also upregulate B7-H1, the ligand of programmed cell death receptor 1 (PD-1), permitting MSCs to inhibit the proliferation, plasma cell differentiation, and IgG secretion of B-cells by direct cell–cell interaction [44].

### 2.2. Different Origins and Types of B-Cells

B-cells of various origins, including rare subpopulations (such as regulatory B-cells (Bregs)), abnormal B-cells from patients with hematological system diseases, precursor B-cells and mature B-cells (the pathways that regulate the transition from mature B-cells to plasma cells or memory B-cells are not reviewed in this section) play different roles in the regulation of MSCs. In particular, CD5-positive B-cells are a peculiar subpopulation with a remarkable immunoregulation ability to maintain peripheral tolerance by secreting IL-10 or inducing the differentiation of T regulatory cells [45,46,47]. Patients with chronic GVHD (cGVHD) have been shown to have impaired CD5+ B-cell reconstitution [48,49].

ASCs from both healthy subjects and breast cancer donors can promote the proliferation of lymphoblastoid Namalva cells (in both standard growth medium and growth factor-deficient medium) and the myeloma U266 cell line. In addition, the production of IgM and IgE is not affected by ASCs in these co-culture systems [50]. BMMNCs from a B-cell acute lymphocytic leukemia (B-ALL) donor (B-ALLBMMNCs) express specific surface markers, including CD19, CD34, terminal deoxynucleotidyl transferase markers (TdT), and CD10, but not CD20. Thus, B-ALLBMMNCs can be considered to be abnormal B-cells. After co-culture with MSCs, B-ALLBMMNCs overexpress CD19, CD10, and CD20 (the expression levels of both CD10 and CD20 increase by a wide margin). Hierarchical cluster analysis of these surface markers shows that, after co-culture with MSCs, an association between pre-pre-B-cells from control patients (Ct) and B-ALLBMMNCs gradually forms. However, no association between these cell groups has been observed after their co-culture in the absence of BMMSCs [51].

MSCs can also promote the proliferation of CD5+ B-cells in a positive feedback manner. MSCs accelerate the survival and proliferation of CD5+ B-cells by secreting IDO. Interestingly, IFN-γ, secreted by CD5+ B-cells, can compound the effects of MSCs [52]. In addition, B7-H1, secreted by MSCs, is indispensable for the upregulation of CD5+ B-cells [37].

### 2.3. MSCs Co-Cultured with B-Cells in Basic Conditions

Here, MSCs and B-cells co-cultured under basic conditions are defined as unstimulated, healthy BMMSCs or ASCs that are co-cultured with stimulated, mature B-cells. Although these basic conditions are different than the microenvironment *in vivo*, this experimental setup is highly stable and repeatable, making it useful for exploring the molecular signaling pathways underlying the interaction between B-cells and MSCs.

According to Healy *et al.*, MSCs can inhibit the caspase 3-mediated apoptosis of peripheral CD19+ B-cells through direct cell–cell interaction. This inhibition is dependent on the MSC-induced upregulation of vascular endothelial growth factor (VEGF), and a p-PKBA blocking experiment showed that the signal transmission following cell–cell contact is not dependent on CXCR12-CXCL4 or epidermal growth factor receptor (EGFR) [53]. ASCs can also secrete an unknown soluble factor that promotes the chemotaxis and mobility of B-cells. However, potential B-cell chemokines, such as PGE2, CXCL8, CXCL10, or combinations of cytokines secreted by ASCs, do not affect on B-cell chemotaxis [54].

**Figure 1:**
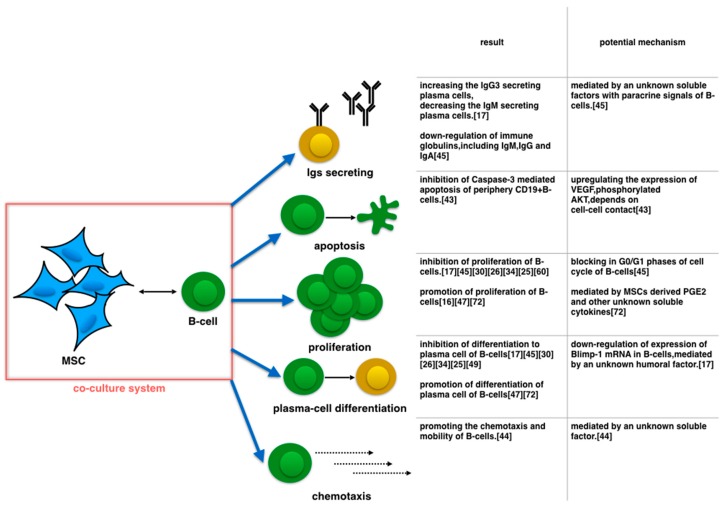
When MSCs are co-cultured with B-cells in basic condition, MSCs regulate Igs-secreting, apoptosis, proliferation, plasma-cell differentiation and chemotaxis of B-cells. For indefinite reasons, such as diverse stimulators of B-cells in culture medium, regulations of proliferation, plasma-cell differentiation and Igs secreting of B-cells are unable to reach agreement.

According to Asari *et al.*, MSCs can inhibit the proliferation of B-cells (stimulated by LPS), and the degree of suppression is related to the MSC–B-cell ratio in the co-culture system. This phenomenon suggests that the B-cell apoptosis induced by MSCs is unrelated to the decrease in total B-cells. In this study, MSCs suppressed LPS-stimulated B-cells, which then could differentiate into plasma cells and affect the proportion of the plasma cell population by decreasing the IgM-secreting plasma cells and instead boosting the IgG3-secreting plasma cells [17]. In addition, the downregulation of Blimp-1 mRNA expression in B-cells resulted in the suppression of B-cell terminal differentiation. This suppression was likely mediated by an unknown humoral factor released by the MSCs (not IL-10, TGF-β, or IDO) [17]. BMMSCs can inhibit the proliferation of B-cells by blocking the G0/G1 phases (G0 phase to G1 phase) of the cell cycle. Interestingly, the supernatants from the purified BMMSCs culture medium cannot suppress the proliferation of B-cells. We suggest that BMMSCs secrete the soluble inhibitory factors stimulated only by the paracrine signals [55]. When B-cells are co-cultured with BMMSCs, several key immunoglobulins and chemokine receptors are down regulated. These molecules include the immunoglobulins IgM, IgG, and IgA and the chemokine receptors CXCR4, CXCR5, and CCR7. However, neither costimulatory molecules (CD40, CD86, and CD80) nor cytokines (TNF, IFN-γ, IL-4, IL-10, and IL-12) expressed by B-cells are affected by MSCs [55]. Interestingly, several reports indicate that MSCs can promote the proliferation and differentiation of B-cells in basic co-culture systems [16,56,57], with MSC-derived PGE2 and other unknown soluble cytokines mediating the positive immunoregulation [58] (Figure 1).

## 3. Influence of Other Immune Cells on MSC–B Cells Crosstalk

A substantial amount of research has demonstrated that different B-cell states and functions are regulated by other immune cells. For example, the activity of B-cells is dependent on antigen presenting cells, such as DCs and helper T cells (Th). After receiving an activation signal, B-cells proliferate and differentiate into plasma-cells [18]. As previously mentioned, MSCs can modify the function of other immune cells through cell–cell interaction or by secreting soluble factors [6,10,11,12,13]. The regulation of Th-cells or DCs by MSCs can greatly influence the interaction between MSCs and B-cells in mixed immune cell environments. MSCs can inhibit the maturation, migration, antigen presentation and cytokine secretion of DCs [12,57,59] via soluble factors, and alter the subpopulation polarization of Th-cells (Th1-Th2) as well as suppress Th-17 differentiation [10,60,61].

### 3.1. In Vitro

Compared with the microenvironment *in vivo*, *in vitro* culture systems provide more stable and controllable surroundings. The experimental results from animal models or clinical trials need to be verified via *in vitro* tests. The co-culture systems commonly used to validate the immune regulation between MSCs and B-cells are MSC–PBMC [62], MSC–B-cell–T-cell [56], and MSC–B-cell–DC systems [63].

Functional T-cells are crucial for B-cell inhibition. When peripheral blood lymphocytes (PBLs) are co-cultured with MSCs and B-cells, MSCs can inhibit B-cell proliferation, plasma cell differentiation, and Ig excretion. However, in the absence of CD3+ cells, this inhibition effect disappears, and MSCs instead promote the proliferation and Ig secretion of B-cells. To confirm this finding, MSCs, B-cells and T-cell were co-cultured in the presence of CpG. In the system, the ability of MSCs to restrain B-cell function was restored. Furthermore, MSCs–T-cell (Th cell) contact is crucial for the inhibition of B-cell proliferation, and an unknown soluble factor from T-cells or MSCs mediates the suppression of B-cells [56]. Interestingly, the content of IFN-γ in the supernatant showed a remarkable decline in this study. Typically, IFN-γ can be detected only in the B-cell–T-cell co-culture system, not in the supernatant of purified B-cell or T-cell culture systems [56]. IFN-γ, secreted by active T-cells, can promote B-cell maturation and the differentiation of B-cells into plasma cells [64]. Another study showed that the inhibition of B-cell proliferation by MSCs depends on the presence of CD4+ T-cells, but the ability of MSCs to modify the differentiation of B-cells into plasma cells is independent of T-cells and IL-6 [65]. Additionally, whereas IFN-γ is detected in both MSCs–B-cell and MSCs–T-cell–B-cell co-culture systems, only IFN-γ from MSC–T-cell–B-cell co-culture systems had an effect on B-cells [65]. MSCs stimulated with IFN-γ acquire the ability to inhibit T-cell proliferation [66]. Therefore, we hypothesize that T-cell-produced IFN-γ is crucial for the immune suppression of B-cells by MSCs.

DCs can also influence the immunoregulation of B-cells by MSCs. Animal experiments have clearly shown that BMMSCs from Balb/c mice can suppress B-cell proliferation by inhibiting BAFF secretion in DCs [63]. To confirm this result, a murine MSC–DC–B-cell co-culture system was designed and this experiment showed that MSCs can inhibit BAFF secretion in DCs [63]. Furthermore, in a purified MSC–B-cell co-culture system, MSCs promoted the proliferation of B-cells through the secretion of BAFF. However, this positive effect of BAFF on B-cells can be offset by the presence of DCs [39].

Galectin-9 (Gal-9) is a critical factor in mediating the inhibition of B-cells by MSCs. The MSCs, activated by either B-cells or IFN-γ, can inhibit B-cell proliferation and Ig release in peripheral blood mononuclear cells (PBMCs), an effect that is mediated by Gal-9 [61]. Cell–cell contact is essential for Gal-9 to mediate this immunoregulation. Because Gal-9 can also influence the function of Th1 cells, the effect of T-cells on the MSC–B-cell interaction cannot be ignored [61].

Membrane vesicles (MVs) secreted by cells can also mediate the interactions between different immune cells [67]. Based on the site of origin of a particular cell, its structural and biochemical properties can vary broadly [68]. MVs play an important role in intercellular signaling by exchanging mRNA, microRNA, and proteins between cells [58]. MSCs can secrete MVs to regulate B-cell functions. MVs secreted by MSCs can suppress B-cell proliferation and the differentiation of CpG-stimulated B-cells into plasma cells. In addition, MVs can inhibit the biosynthesis of IgM, IgG, and IgA in plasma cells. However, MVs do not facilitate B-cell apoptosis. Furthermore, MVs typically contain IL-6 and IL-8, and MVs can be endocytosed by B-cells but not by T-cells or NK-cells [69]. Compared with MSCs, secreted MVs are less able to alter the ratio of B-cells and plasma cells in a PBMC population [70].

### 3.2. In Vivo

The allogeneic or autologous transplantation of MSCs shows remarkable therapeutic effects, in both clinical trials and animal models, for many diseases. In conventional cell transplantation therapy, MSCs are injected into humans or animals via a peripheral vein. In the peripheral blood, MSCs can then interact with blood cells other than B-cells. For example, MSCs can also promote the survival and phagocytosis of neutrophils [52,71,72,73] and enhance the phagocytosis of monocytes [70,74]. Importantly, the impact of MSCs on T-cells *in vivo* is reciprocal; T-cells can also immunosuppress MSCs [56]. This immunosuppression can mitigate the action of B-cells in the body, restoring the balance of B-cells, which likely contributes to symptom relief and the improvement of histopathology in autoimmune diseases, GVHD, and certain anaphylactic diseases. Current studies on the mechanism by which MSCs alleviate diseases *in vivo*, via B-cell immune regulation, focus on SLE and cGVHD. *In vivo* studies can be divided into two categories, clinical and animal studies. Compared with clinical tests, the results obtained from mouse or rat models are more reliable and repeatable. The following paragraph summarizes research studies on SLE and cGVHD, from animal models to clinical results.

SLE is a typical multi-system autoimmune disease. The initial burst of SLE symptoms is related to a functional T-cell disorder and the over activation of polyclonal B-cells [24]. As a typical producer of antinuclear antibodies (ANAs), especially anti-dsDNA antibodies, B-cells play a crucial role in the pathophysiologic process of SLE [70]. Recent studies have shown that defects in HSCs or the bone marrow microenvironment can initiate SLE, and MSC transplantation may improve it [75,76]. Recent studies show that regardless of *in vitro* or *in vivo* experiments, MSCs collected from SLE patients or animal models present deficient immune suppression, especially to B-cells [35,77,78]. Based on the deficiency of the immune regulation capacity of BMMSCs from SLE patients or animal models, we can infer that MSCs therapy can alleviate SLE by inhibiting the excessive proliferation of B-cells and secretion of autoantibodies in the bone marrow niche and peripheral blood. Does the therapeutic effect of MSCs appear in SLE animal models? Studies indicate that it does. To investigate the therapeutic effects and mechanism of MSCs, several murine models have been utilized, such as *MRL/lpr* mice [66], *(NZB/NZW)F1* mice [78] and *Roquinsan/san* mice. The results of MSC therapy on SLE murine models show that the therapy: (1) prolongs survival time [48,58]; (2) reduces proteinuria and glomerulonephritis [65,70,79]; (3) lightens the spleen [58]; (4) suppresses serum anti-dsDNA antibodies and the deposition of immune complexes [35,66,78,79]; and (5) reduces serum immune globulin (IgG, IgE, and IgM) [79]. The immune regulation of the B-cell subpopulation in SLE models receiving the therapy indicates that MSCs reduce germinal center B-cells [79], plasma cells [79], follicle B-cells [79] and naive mature (CD19+ and CD21+) B-cells and promote the marginal zone B-cells [79] and regulatory B-cells [79]. Regarding the functions of B-cells, MSCs suppress the maturation, plasma-cell differentiation and antibody (IgM and IgG) secretion of B-cells [66,79]. Additionally, MSCs restore the bone marrow niche microenvironment [47]. In general, MSCs therapy shows a positive effect on SLE murine models.

Can clinical tests, duplicate the positive effects on animal models? Clinic tests involving MSC transplantation in SLE patients are limited in number, but the curative effects are valid. Reduction of the systemic lupus erythematous disease activity index (SLEDAI) [6,47,80], recovery of renal function [47,81], and a decrease in serum anti-dsDNA antibodies and compliment C3 [47,80] are observed in recipients of MSCs therapy. However, data pertaining to the immune regulation of MSCs to B-cells in clinical tests are lacking.

GVHD is typically initiated after allogeneic hematopoiesis. cGVHD has become one of the most common serious problems affecting long-term hematopoietic stem cell transplantation (HSCT) survivors [81,82]. cGVHD has long been considered to be an autoimmune disorder, and medicines used to treat cGVHD remain unsatisfactory, particularly for refractory cGVHD [83]. Recently, quite a few studies have demonstrated that not only T-cells but B-cells as well play a vital role in the process of cGVHD [84]. Excessive serum BAFF and insufficient naive B-cells can be observed in cGVHD patients. The coexistence of the two anomalies activates autoimmune B-cells in patients, thus leading to autoimmune symptoms [85]. Inhibiting B-cells to a certain degree in peripheral blood and activating Bregs after HSCT are beneficial to preventing cGVHD [86,87,88]. Because studies show that MSCs possess the ability to immunosuppress B-cells, inhibit DCs secreting BAFF and up-regulate Bregs, MSC therapy is chosen as a potential option for relieveing cGVHD. In GVHD animal models, the outcomes of MSC therapy are contradictory. The curative effect is dose-dependent and related to the timewindow of MSC transplantation [88,89]. If MSC transplantation is implemented while HSCT is in progress, MSCs decrease the morbidity of cGVHD [89], prolong survival time [88,90], reduce the cGVHD score [88,90] and decrease the serum IFN-γ [65]. However, another group reports that MSC therapy exhibits a negligible effect in preventing GVHD [91]. Once GVHD is initiated, MSC injection cannot alleviate symptoms or target organ damage [89].

The inconsistent outcomes of MSC therapy in animal models are bewildering. To gain more meaningful and practical results, clinical tests of MSC transplantation on GVHD patients have been designed and carried out. Small-scale clinical tests show that MSC therapy alleviates damage to target organs (liver, skin, gut, and oral mucosa) [52,92], but its effects on overall survival or the rate of complete remission are controversial [42,92,93,94]. For glucocorticoid-resistant patients, the therapeutic effect is invalid [92]. However, for less heavily treated patients, MSC transplantation shows a positive curative effect [93,94]. The relevance of glucocorticoid therapy and the effects of MSC transplantation should be investigated further. In the peripheral blood of complete remission patients, as the frequency of Bregs increases, the IL-10 secretion of Bregs is up-regulated [95]. The mechanism of Bregs immune regulation of MSCs is related to down-regulating of the serum BAFF and up-regulating of the ratio of naive B-cells and the memory B-cells [95].

## 4. Concluding Points and Future Directions: Do IL-4 and LPS Play a Role?

The interaction between MSCs and B-cells is sophisticated; diverse culture environments and types of cells affect the immunoregulation of B-cells by MSCs. In purified B-cell–MSC environments, MSCs regulate B-cell functions via soluble factors (Table 1) and cell–cell contact. We found that the soluble factors related to the immunoregulation of B-cells can be divide into two subtypes based on their function: pro-inflammatory and anti-inflammatory. Membrane vesicles, CCL2, B7-H1, VEGF, C3, GAL-9, and IDO are anti-inflammatory, whereas BAFF, PGE2, and APRIL are pro-inflammatory. In the co-culture systems listed above, the B-cell stimulators and the ratio between MSCs and B-cells were varied, yet MSCs were still able to modulate the functions of B-cells. Therefore, this process likely depends on paracrine signaling [96]. We hypothesize that MSCs are influenced by diverse stimulators in co-culture systems, which can lead to the distinct immune regulation of B-cells by MSCs observed in different experimental setups.

Several other types of immune cells can differentiate into two subpopulations with contrasting functions depending on the signals in the microenvironment. For example, Th-cells can differentiate into the Th1 and Th2 subpopulations [97] and macrophages can differentiate into the M1 and M2 subpopulations [98]. In both of these cases, one of the subpopulations is pro-inflammatory, whereas the other is anti-inflammatory. The induction, progress and recovery of inflammation in the human body relies on the self-balancing of pro- and anti-inflammatory signals and cell types. Interestingly, MSCs possess characteristics similar to those of these immune cells. MSCs can transform into a pro-inflammatory MSC1 subpopulation or an anti-inflammatory MSC2 subpopulation, depending on the microenvironment [99,100]. Recent studies have reported that when different TLRs subtypes on MSCs are stimulated, MSCs can differentiate into distinct subgroups. For example, stimulation of TLR4 is indispensable for MSC1 differentiation and TLR3 is indispensable for MSC2 differentiation [99]. We can speculate that the MSC–B-cell co-culture systems that led to an inhibition of B-cells likely contained TLR3 stimulators (poly(I:C), *etc.*) or anti-inflammatory differentiation-related factors (IL-4, IL10, and TGF-β), and the co-culture systems that promoted B-cells contained TLR4 stimulators (LPS, *etc.*).

In purified B-cell–MSC co-culture systems, the regulation of B-cells does not remain uniform, particularly for the regulation of B-cell proliferation and plasma-cell differentiation. To confirm the above-mentioned hypothesis, we examined the different B-cell stimulators in each culture medium (Table 2). Interestingly, with the exception of one study, we observed that all of the co-culture systems in which B-cells were suppressed contained IL-4 or LPS. However, why would LPS, a TLR4 stimulator, lead to B-cell suppression? Existing research suggests that long-term and excessive LPS stimulation induces macrophages to switch to the anti-inflammatory M2 subpopulation. MSCs are likely stimulated by LPS in certain co-culture systems, thus leading to the suppression of B-cells.

Although the immunoregulation of B-cells by MSCs is largely mediated by soluble factors, cell–cell contact also plays a role in the MSC–B-cell interaction. Similar to immune suppression mediated by T-cells [101,102], cell–cell contact between MSCs and B-cells is essential for facilitating B7-H1 and Gal-9 to inhibit B-cell function [44,61], promote the survival and IgG secretion of B-cells [63,103], and suppress caspase-3-mediated B-cell apoptosis [53].

In *in vitro* environments with multiple types of immune cells, MSCs typically inhibit B-cell proliferation and plasma-cell differentiation [56,61,64,66,69,70] and promote the proliferation of Bregs [37,72]. The immunosuppression of MSCs is related to specific cytokines, such as IFN-γ and BAFF. After receiving paracrine signals from B-cells, T-cells secrete IFN-γ, and this IFN-γ-secreting capacity of T-cells can, in turn, be suppressed by MSCs.

Therefore, MSCs can inhibit B-cell proliferation and plasma-cell differentiation through inhibiting IFN-γ secretion in T-cells. Similarly, MSCs can inhibit B-cell through the downregulation of BAFF secreted by DCs. *In vivo*, in both human and animal experiments, MSCs can suppress abnormal proliferation and excess autoantibody production of B-cells through two mechanisms. First, injected MSCs can substitute for the dysfunctional MSCs in patients or animal models and regulate the aberrant proliferation and antibody secretion of B-cells. Second, MSCs can be stimulated by other immune cells and become anti-inflammatory; they can then suppress B-cells and instead promote the proliferation of Bregs. Several questions remain to be answered in the field of stem cell biology: (1) Which soluble cytokines can switch the immune regulation of MSCs (from promotion to inhibition)? (2) How do the paracrine signals from B-cells influence the immunoregulation of MSCs? (3) How can we design *in vitro* MSC co-culture systems such that we can simulate the peripheral blood of a patient with a specific autoimmune disease to investigate why MSCs regulate B-cells differently *in vivo*? (4) How can we build an MSC culture system to accurately promote MSC subgroup (M1 or M2) differentiation to an appropriate extent to optimize the MSC therapy? Answering these questions will contribute to finding cytokines suited for stimulating MSCs to rectify the immune regulation of B-cells for specific clinical applications (e.g., to relieve a diverse range of autoimmune diseases and identify triage patients fit for treatment with MSCs by assaying the cytokines in their peripheral blood).

## Figures and Tables

**Table 1:** Soluble factors related to the interaction between MSCs and B cells.

Soluble Factor	Original Mscs	The Effect of B-Cells	Explanatory Note
Unknown Factors [17,40,54,55]	Human UC-MSCs [40] Murine BM-MSCs [17] Human BM-MSCs [55] Human ASCs [54]	proliferationβ ↓ [17,40,55]	
		plasma-cell differentiation ↓ [17,40,55]	
		Ig secretion ↓ [17,40,55]	
		apoptosis ↓ [17]	
		chemotaxis ↓ [54]	
Membrane Vesicles (Containing IL-6 AND IL-8) [69,70]	Human BM-MSCs [69,70]	proliferation ↓ [69,70]	the immune suppression of purified IL-6 or IL-8 to B-cells has not been confirmed.
		plasma-cell differentiation ↓ [69,70]	
		Ig secretion ↓ [69]	
		Pro-inflammatory Cytokines secretion ↓ [70]	
		Anti-inflammatory Cytokines secretion↑ [70]	
CCL2 [35,36]	Human BM-MSCs [35,36]	proliferation ↓ [35,36]	
		plasma-cell differentiation ↓ [36]	
		Ig secretion ↓ [35,36]	
B7-H1 [44]	Murine BM-MSCs [44]	proliferation ↓ [44]	
		Ig secretion ↓ [44]	
VEGF [53]	Human BM-MSCs [53]	Apoptosis ↓ [53]	
Complement C3 [42]	mycoplasma-infected MSCs [42]	Ig secretion ↓ [42]	
GAL-9 [58]	Human BM-MSCs [58]	proliferation ↓ [58]	
		Ig secretion ↓ [58]	
IDO [52]	Human BM-MSCs [52]	breg proliferation ↑ [52]	
		Anti-inflammatory Cytokines secretionα ↑ [52]	
BAFF [38,39]	Human BM-MSCs [39]	proliferation ↑ [38,39]	
	Murine BM-MSCs [39]		
	adipogenic differentiated MSCs [38]		
PGE2 [18,43]	Human UC-MSCs [18]	proliferation ↑ [18]	
	Murine BM-MSCs prepared by TNF-α [43]	Ig secretion ↑ [18]	
		IgE secretion ↓ [43]	
APRIL[41]	Human CLLBM-MSCs [41]	proliferation ↑ [41]	the immunoregulation of APRIL to B-cells is a conjecture. *in vitro* experiment to clarify has not carried out.
		Ig secretion ↑ [41]	
C/EBPΒ[37]	Murine BM-MSCs [37]	precursor B-cell proliferation ↑ [37]	

Remarks: ↑, promoting; ↓, suppressing.

**Table 2:** Correlation between stimulators in culture media (LPS, CpG, CD40L, and Ig antibodies IL-2 and IL-4) and the immunoregulation of B-cells by MSCs.

MSCs	B-Cells	LPS	CpG	CD40L	Ig Antibodies	IL-2	IL-4	Proliferation of B-Cells	Plasma-Cell Differentiation of B-Cells
murine BMMSCs	murine B-cells	√						↓ [17]	↓ [17]
human BMMSCs	human B-cells		√	√	√	√	√	↓ [55]	↓ [55]
human UCMSCs	murine B-cells		√	√	√		√	↓ [40]	↓ [40]
human BMMSCs	mutine B-cells		√	√	√		√	↓ [36]	↓ [36]
murine BMMSCs	murine B-cells	√					√	↓ [44]	↓ [44]
human BMMSCs	human B-cells		√	√	√		√	↓ [35]	↓ [35]
human UCMSCs	human B-cells		√	√	√	√		↓ [83]	
human BMMSCs	human B-cells		√	√	√	√		↓ [83]	
human ASCs	human B-cells			√	√	√		↛ [65]	↓ [65]
human BMMSCs	human B-cells		√	√				↑/↛(FCS/PL) [16]	
human ASCs	human B-cells		√	√				↑ [16]	
human UCMSCs	human B-cells		√	√	√	√		↑ [18]	↑ [18]
human BMMSCs	human B-cells		√					↑ [56]	↑ [56]

Remarks: ↑, promoting; ↓, suppressing; ↛, no effect.

## Abbreviations

MSCs, mesenchymal stem cells; NK-cells, natural killer cells; DCs, dendritic cells; SLE, systemic lupus erythematous; GVHD, graft-versus-host disease; IBD, inflammatory bowel disease; SS, systemic sclerosis; IL, interleukin; TGF-β, transforming growth factor-β; PGE2, prostaglandin E2; NO, nitric oxide; IDO, indoleamine 2,3, dioxygenase; LMSCs, lupus mesenchymal stem cells; CpG, cytosine phosphorothioate guanine; sCD40L, soluble cluster of differentiation 40 ligand; Ig, immunoglobulin; CCL2, C-C-motif ligand 2; MMP, matrix metalloproteinase; OAZ, olfactory 1/early B cell factor-associated zinc-finger protein; BMMSCs, bone marrow mesenchymal stem cells; C/EBPβ, CCAAT/enhancer-binding protein β; SDF-1, stromal cell-derived factor 1; BAFF, B-cell activating factor; TLR4, Toll-like receptor 4; UCMSCs, umbilical cord mesenchymal stem cells; Blimp-1, B-cell-induced maturation protein-1; PAX-5, paired box gene 5; CLLBMMSCs, B-cell chronic lymphoblastic leukemia; APRIL, a proliferation-inducing ligand; SDF-1, stromal cell-derived factor 1; LPS, lipopolysaccharide; C3, complement C3; IFN-γ, interferon-γ; TNF-α, tumor necrosis factor-α; ASC, mesenchymal stem cell from adipose tissue; adi-MSCs, mensenchymal stem cells differentiated into adipocytes; PL, human platelet lysate; FCS, fetal calf serum; PD-1, programmed cell death 1; Bregs, regulatory B cells; HSCs, hematopoietic stem cells; cGVHD, chronic graft-versus-host disease; B-ALLBMMNCs, B-cell acute lymphocytic leukemia bone marrow mononuclear cells; TdT, terminal deoxynucleotidyl transferase markers; Ct, control patients; VEGF, vascular endothelial growth factor; EGFR, epidermal growth factor receptor; CXCL, C–X–C-motif ligand; Gal-9, galectin-9; PBMCs, peripheral blood mononuclear cells; MVs, membrane vesicles; ANA, antinuclear antibodies; NF-κB, nuclear factor kappa-light-chain-enhancer of activated B cells; MAPK, mitogen-activated protein kinase; JNK, c-Jun N-terminal kinases; p-p38, phosphorylated protein-38; p-PKB, phosphorylated Protein kinase B; COX2, cyclo-oxygen-ase 2; SLEDAI, systemic lupus erythematous disease activity index.
